# The Need to Introduce Simulation-Based Teaching in Pharmacy Education in Saudi Arabia

**DOI:** 10.3390/pharmacy6030060

**Published:** 2018-07-03

**Authors:** Ejaz Cheema

**Affiliations:** 1 College of Pharmacy, Umm-Al-Qura University, Taif Road, Makkah 13578, Saudi Arabia; E.cheema.1@warwick.ac.uk; Tel.: +966-542719203; 2 Warwick Medical School, Warwick University, Gibbet Hill Road, Coventry CV4 7AL, UK

**Keywords:** simulation, pharmacy practice, education, Saudi Arabia

## Abstract

Pharmacists worldwide, including Saudi Arabia, are now increasingly expected to play a more patient-centred role. The transition of pharmacists from a dispensing role to a more patient-centred clinical role requires the adoption of innovative learning techniques in pharmacy teaching and learning to transform the future pharmacy workforce. One such innovation in pharmacy education is simulation-based pharmacy teaching. The use of simulation in pharmacy education allows pharmacy students to not only improve their clinical knowledge and skills, but also serves as a tool to improve their critical thinking that is a pre-requisite in sound clinical decision-making. Given the importance of patient-oriented teaching in pharmacy education, the majority of institutions offering pharmacy education in the developed countries have successfully integrated simulation-based teaching in their respective curricula to meet both patient and practice needs. However, most of the universities offering undergraduate pharmacy programs in the developing world, including Saudi Arabia, have limited application of patient-focused teaching in their respective programs. This article aims to highlight the importance of introducing simulation-based teaching in pharmacy education in Saudi Arabia.

## 1. Introduction

Pharmacists worldwide are now increasingly expected to play a more patient-centred role. For example, in the United Kingdom (UK), the White paper titled “A vision for pharmacy in the new National Health Service” (NHS) outlined the UK government’ vision for community pharmacy [1]. It called for utilizing pharmacy strengths towards the delivery of a safer, effective and more patient-centred care. The introduction of the new community pharmacist contract in 2005 was the first step that attempted to move pharmacists towards a more clinically oriented role. Since the introduction of the new community pharmacist contract in the UK, many patient-focused pharmacy services were introduced including the Medicines Use Reviews (MURS) and the New Medicines Service (NMS). More recently, new models of pharmacist-led patient care have emerged in the UK that call for the delivery of a patient-centred, value-driven and outcome-based approach to medicines use [2]. Pharmacists all over the UK are being deployed in medical practices to support the management of long-term medical conditions [2,3]. Similarly, in the USA, pharmacists have the opportunity to be part of the multi-disciplinary teams that provide services to the recently introduced patient-centred medical homes (PCMHs) [4].

The transition of pharmacists from a dispensing role to a more patient-centred clinical role requires the adoption of innovative learning techniques in pharmacy teaching and learning to transform the future pharmacy workforce. One such innovation in pharmacy education is simulation-based pharmacy teaching. Simulation has been defined as ‘(an) event or situation made to resemble clinical practice as closely as possible’ [5]. The use of simulation in pharmacy education allows pharmacy students to not only improve their clinical knowledge and skills, but also serves as a tool to improve their critical thinking that is a pre-requisite in sound clinical decision making [6]. The use of ‘standardized patients’ in simulation allows students to apply their clinical knowledge to inpatient settings while minimizing the risk to patients [7]. It may be argued that the use of standardized or controlled patients in the simulation may limit students’ ability to learn patient empathy. However, a pilot study that assessed the impact of simulation on the clinical skills and knowledge of nursing students in the USA suggested that provision of simulation-based teaching coupled with clinical experience led to an improvement in the clinical knowledge and skills of students [8]. Similarly, another study that aimed to determine the impact of simulation on blood pressure measurement skills of pharmacy students enrolled in a Doctor of pharmacy program (PharmD) reported significant improvement in the clinical skills of students [9]. 

Given the importance of patient-oriented teaching in pharmacy education, the majority of institutions offering pharmacy education in the developed countries have successfully integrated simulation-based teaching in their respective curricula to meet both patient and practice needs. For example, the council responsible for accrediting pharmacy education in the USA has approved up to 20% use of simulation in the practical component of pharmacy practice education [10]. However, most of the universities offering undergraduate pharmacy programs in the developing world including Saudi Arabia have limited application of patient-focused teaching in their respective programs [11]. This article aims to highlight the importance of introducing simulation-based teaching in pharmacy education in Saudi Arabia.

## 2. Importance of Simulation-Based Education in Pharmacy from Saudi Arabia’s Perspective

Pharmacy education in Saudi Arabia started in 1959 for the first time with the introduction of a four-year bachelor of pharmacy and medicinal chemistry program [12]. The initial four-year degree was then later modified both by the inclusion of new courses and by extending the length of a degree to five years [13]. The year 2005 witnessed the introduction of a six-year PharmD program in Saudi Arabia which laid greater emphasis on pharmacy practice and clinical pharmacy courses than in the traditional Bachelor of pharmacy (BPharm) program. At present, 28 universities including both private and government are offering BPharm and PharmD programs in Saudi Arabia [14] with some universities offering both programs to pharmacy students. However, the clinically-oriented PharmD program seems to be more popular among the Saudi students.

Although the academics in Saudi Arabia intend to align the PharmD curriculum on the lines of PharmD program offered in Canada and USA, this alignment is not possible without addressing some of the barriers to successful implementation of PharmD program in Saudi Arabia. Some of these challenges include lack of qualified clinical faculty coupled with a limited number of hospital training sites [14] and non-standardized clinical training. PharmD students have to complete mandatory 12-month clinical rotations in their sixth year as part of their degree. These clinical rotations must be completed under the supervision of preceptors. Provision of clinical training to a growing number of PharmD students is becoming a challenge for universities as most do not have teaching hospitals. In the absence of limited training sites and qualified clinical preceptors, students often have to be rotated between different hospital sites to ensure the provision of training to all students. Furthermore, the quality of clinical training provided to students is also variable across the hospitals. Although a few large governmental hospitals including the King Faisal specialist hospital and research center, National Guard Hospital and King Abdullah Medical City have a structured and advanced clinical training program, they have a limited number of training positions for students. Many other hospitals and primary healthcare centers neither have a structured training program, nor qualified clinical preceptors. With such inconsistency in the quality of training, provision of standardized clinical training to all students cannot be guaranteed. 

Simulation-based clinical teaching can address some of these challenges. Simulation-based labs can accommodate a maximum number of students without the fear of slashing the number of students’ intake in the training program. The controlled and risk-free environment for patients in simulation labs allow students to work on different patient scenarios that may not be presented to them during their traditional clinical placement. Furthermore, simulation labs led by qualified faculty can also ensure the delivery of a standardized training program to all students. 

## 3. Conclusions

The clinically oriented PharmD program in Saudi Arabia promises to open new horizons for future pharmacists by allowing them to play an active role in the expanding healthcare system. However, successful implementation of the PharmD program in Saudi Arabia faces many challenges. Given the importance and utility of simulation-based teaching in pharmacy practice, academic institutions in Saudi Arabia should move towards integrating simulation in their curricula to overcome these challenges and to better prepare the future pharmacy workforce in the current era of constant change.
